# Onnamides and a Novel Analogue, Onnamide G, as Potent Leishmanicidal Agents

**DOI:** 10.1007/s10126-025-10494-1

**Published:** 2025-09-05

**Authors:** Takahiro Jomori, Nanami Higa, Trianda Ayuning Tyas, Natsuki Matsuura, Yudai Ueda, Ayumi Suetake, Shin Miyazaki, Shuichi Watanabe, Sei Arizono, Yasuhiro Hayashi, Ko Yasumoto, Yuji Ise, Toshiyuki Wakimoto, Mina Yasumoto-Hirose, Junichi Tanaka, Kanami Mori-Yasumoto

**Affiliations:** 1 Department of Chemistry, Biology and Marine Science, Faculty of Science, University of the Ryukyus, Nishihara, Okinawa 903-0213 Japan; 2 Faculty of Pharmaceutical Sciences, Tokyo University of Science, Niijuku, Tokyo, Katushika 125-8585 Japan; 3 Faculty of Agriculture, University of Miyazaki, Gakuen-Kibanadai-Nishi, Miyazaki, Miyazaki 889-2192 Japan; 4 School of Marine Biosciences, Kitasato University, Kitasato, Minami, Sagamihara, Kanagawa 252-0373 Japan; 5 Faculty of Human Environmental Studies, Hiroshima Shudo University, Ozuka, Asaminami-ku, Hiroshima, 731-3195 Japan; 6 Faculty of Pharmaceutical Sciences, Hokkaido University, Sapporo, Hokkaido 060-0812 Japan; 7 Tropical Technology Plus, Uruma, Okinawa 904-2234 Japan

**Keywords:** Leishmaniasis, Antileishmanial activity, *Leishmania major*, *Theonella* sp., *Discodermia irregularis*, Onnamides, Cytotoxicity

## Abstract

Leishmaniasis is a neglected tropical disease posing significant global health challenges. Given the limited effective treatment options and associated constraints, many patients are unable to complete therapy. In this study, we report the remarkable leishmanicidal activity of onnamides derived from the marine sponge *Theonella* sp. against the promastigote form of *Leishmania major* (IC_50_ = 0.2–140 nM) and demonstrate their selectivity through cytotoxicity assessments on human hepatoma HepG2 cells. Structural-activity relationship (SAR) analyses were conducted using 6,7-dihydro-onnamide A and its analogs. Furthermore, we compared the action mechanisms of amphotericin B, the primary drug currently used for treatment, and the obtained onnamides. Based on biological evaluations and SAR studies, onnamides can be considered promising candidates for anti-leishmanial chemotherapy, warranting further investigation.

**Supplementary Information:**

The online version contains supplementary material available at 10.1007/s10126-025-10494-1.

## Introduction

Leishmaniasis is a parasitic disease endemic to 90 tropical and subtropical countries, affecting approximately 12 million people, with 350 million at risk (Zhang et al. 2025). It manifests in three forms: cutaneous, mucosal, and visceral, with cutaneous leishmaniasis (CL) being the most common, causing skin sores that can result in severe scarring and social stigma. Current treatments, including antimonial compounds, liposomal amphotericin B, and miltefosine, face major challenges such as high toxicity, side effects, high costs, and drug resistance. Additionally, the lack of vaccines and improved diagnostics highlights the urgent need for safer and more accessible therapies, particularly for patients in endemic regions.

Onnamides are a key subgroup of polyketide-containing nitrogenous compounds, initially reported by Sakemi et al. in 1988 and sourced from the marine sponge *Theonella* sp. collected at Manza, Okinawa, Japan (Sakemi et al. 1988). Onnamides have also been isolated from marine sponges of the genera *Theonella* in Hachijo island (Matsunaga et al. 1992) and Amami island (Nakamura et al. 2023) of Japan, *Discodermia* spp. from Jeju island of Korea (Shinde et al. 2007), and Honduras (Paul et al. 2002) and *Trachycladus* spp. in Southern Australia (Vuong et al. 2001). They are biosynthesized via a polyketide and nonribosomal peptide hybrid pathway and feature a core tricyclic structure of two interconnected tetrahydropyran rings linked by an *N*-acyl aminal moiety (Mosey and Floreancig 2012). Onnamides are characterized by an arginyl amino acid residue connected to an unsaturated fatty acid chain. Although onnamide A has shown limited potency in vivo due to restricted cellular diffusion of its charged guanidino group, onnamide A and its derivatives exhibit strong in vitro anticancer activity. Studies have demonstrated potent antitumor activity against various cancer cell lines, with IC_50_ values ranging from nanomolar to low micromolar range (Kobayashi et al. 1993; Matsunaga et al. 1992; Nakamura et al. 2023). These findings suggest that such compounds are promising candidates for further development as antitumor agents.

**Fig. 1:**
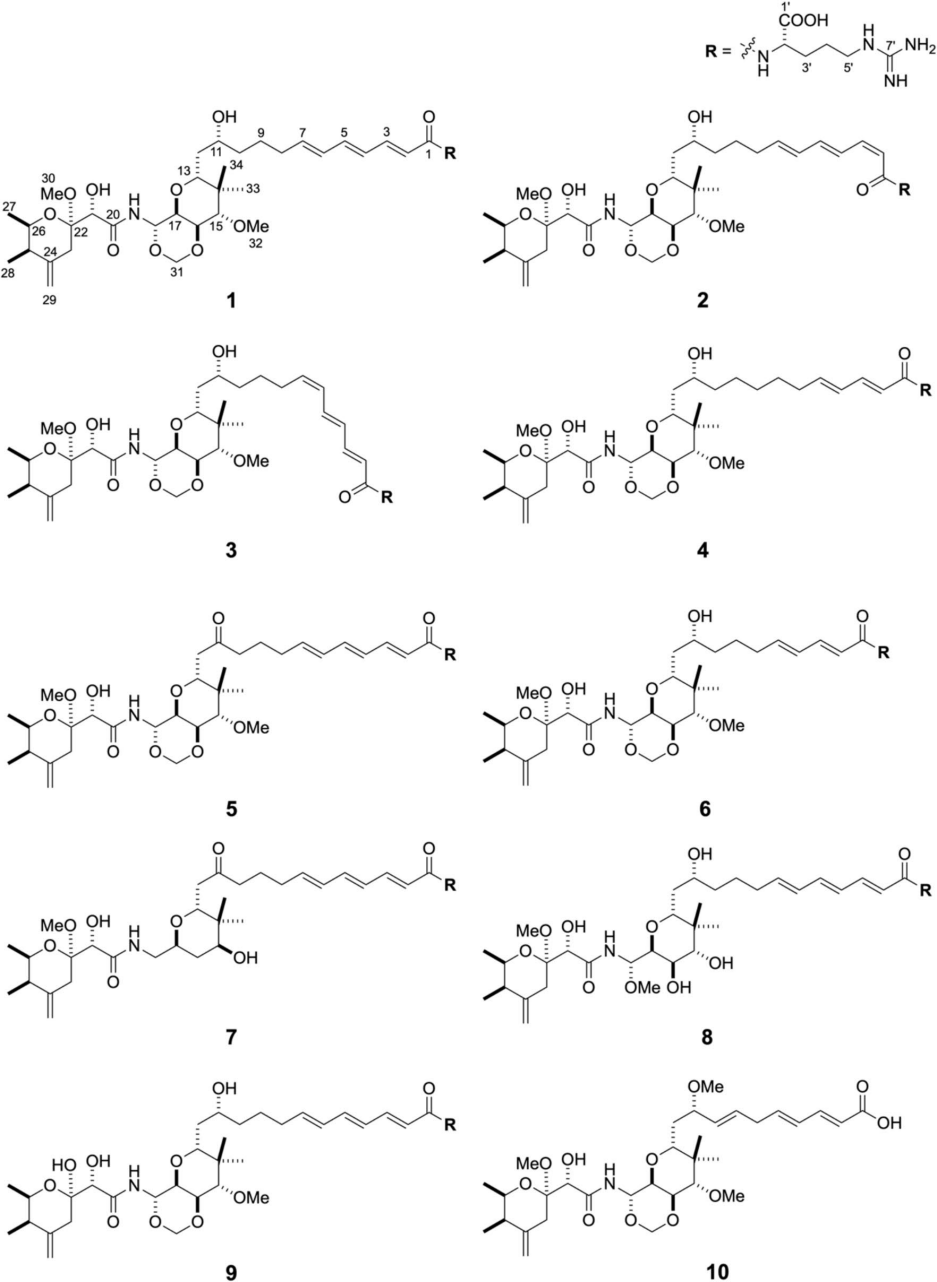
Structures of isolated compounds **1**–**10**

During the course of our search for potential drug candidates against leishmaniasis (FY2022-2024 Okinawa Innovation Ecosystem Joint Research Promotion Project), the methanol extract of *Theonella* sp. (yellow interior) collected at Manza in 2023 demonstrates > 90% growth inhibition of that of the promastigote *Leishmania major* at 10 µg/mL. Through bioassay-guided fractionations using open-column chromatography and reversed-phase HPLC, we isolated onnamide A (**1**) (Sakemi et al. 1988), 2*Z*-onnamide A (**2**) (Nakamura et al. 2023), 6*Z*-onnamide A (**3**) (Nakamura et al. 2023), 6,7-dihydro-onnamide A (**4**) (Matsunaga et al. 1992), 11-oxo-onnamide A (**5**) (Kobayashi et al. 1993),^9^ onnamide B (**6**) (Matsunaga et al. 1992), and onnamide E (**7**) (Matsunaga et al. 1992), combined with a new one named onnamide G (**8**). To gain more insight into the structural-activity relationship (SAR), pseudo-onnamide A (**9**) (Matsunaga et al. 1992), previously isolated from an old methanol extract from *Theonella* sp. collected at the same region in 2012 (Caridha et al. 2019), was also tested for leishmanicidal activity. Another onnamide analogue lacking the arginyl residue, theopederin K (**10**) (Paul et al. 2002), was also isolated from the deep-sea sponge *Discodermia irregularis*. Herein, we report the structural elucidation of onnamide G (**8**), and leishmanicidal activities of ten isolated compounds (Fig. 1).

## Materials and Methods

### General Experimental Procedures

^1^H, ^13^C and 2D NMR spectra were recorded on Bruker Avance III 500 spectrometer (500 MHz for ^1^H NMR,125 MHz for ^13^C NMR). Chemical shifts are denoted in δ (ppm) relative to the residual solvent peaks as internal standards (CDCl_3_, δ_H_ 7.26, δ_C_ 77.0). Data for NMR spectra were reported as follows: chemical shifts (δ) in ppm; s, singlet; d, doublet; t, triplet, m, multiplet; br, broad signal; *J*, coupling constants in Hz. Data were analyzed using Topspin 4.1.4. Organic solvents were purchased from Fujifilm Wako Pure Chemical Corp. ESI–MS spectra were recorded on a Thero Fisher Scientific Orbitrap Exploris 240 spectrometer. Optical rotations were recorded on a JASCO P-1010 polarimeter. All reagents were used as supplied, unless otherwise stated. Column chromatography was performed using silica gel (Cosmosil 75C_18_-OPN); Thin layer chromatography (TLC), silica gel 60 F_254_ plates (Merck). High-performance liquid chromatography (HPLC), Hitachi L-6000 pump fitted with a Hitachi L-4000 UV monitor and a Shodex RI-101 monitor to detect compounds. A 5C_18_-AR-II column (Cosmosil) was used for reversed-phase HPLC. The details of the conditions for HPLC are described below.

The *Leishmania* growth medium consisted of Medium-199 supplemented with 10% fetal calf serum, 100 IU/mL penicillin, and 100 µg/mL streptomycin (M199). The assay reagent for *Leishmania* promastigotes was Cell Counting Kit-8 (Dojindo Molecular Technologies, Inc.).

### Specimen, Extraction, and Isolation of Compounds (1–8)

The fresh marine sponges *Theonella* sp. (yellow interior; 6.5 kg. wet) were collected at Manza, Okinawa, Japan in June 26th, 2024 (26°29′36.0"N 127°50′02.9"E), which was the same sponge identified as *Theonella* sp. in the previous research (Sakemi et al. 1988). The pieces of the tissues were extracted repeatedly with methanol (MeOH), and it was concentrated in vacuo. The MeOH extract was partitioned between ethyl acetate (EtOAc) and H_2_O, the H_2_O layer subsequently subjected onto C_18_-OPN gel (Cosmosil) and eluted with a stepwise gradient of 0%, 20%, 50%, 70% 90%, and 100% MeOH in water. The 70% MeOH fraction, containing onnamides, confirmed by the characteristic peaks around 5.0–7.0 ppm of ^1^H NMR data of onnamides, were further purified by reversed-phase HPLC (Cosmosil 5C_18_-AR-II, 10 mm i.d. × 250 mm, diode array detector (DAD); ranges from 190 to 400 nm; eluted with 55% MeOH heated at 40 °C with column oven) which yielded onnamide G (**8**, 0.5 mg), onnamide B (**6**, 13.5 mg), onnamide E (**7**, 1.0 mg), onnamide A (**1**, 110.5 mg), fraction A (21.2 mg), and fraction B (35.1 mg). The fraction A was separated by reversed-phase HPLC (Cosmosil 5C_18_-AR-II [10 mm i.d. × 250 mm], eluted isostatically with 35% acetonitrile in water heated at 40 °C with column oven) to give 11-oxo-onnamide A (**5**, 2.5 mg) and fraction C (6.9 mg). The fraction C was further purified by RP HPLC (Cosmosil, πNAP, 10 mm i.d. × 250 mm, eluted with 33% acetonitrile in water heated at 40 °C with column oven) to isolate 6*Z*-onnamide A (**3**, 1.2 mg), 2Z-onnamide A (**2**, 0.6 mg). 6,7-Dihydro-onnamide A (**4**, 4.0 mg) was purified with RP-HPLC Cosmosil 5C_18_-AR-II (10 mm id × 250 mm) with 33% acetonitrile in water from the fraction B. Pseudoonnamide A (**9**, 2.9 mg) was used for leishmanicidal assay which was isolated in our previous work (Hayashi et al. 2024).

The sponge *Discodermia irregularis* was collected by dredging at a depth of 175 m at the seamount Ohshima-shinsone, Kagoshima, Japan (28°53′01.2"N 129°33′00.1"E) in June 21, 2019. The marine sponge (173.3 g wet) was kept at −30 °C. The frozen tissue was extracted with acetone for three times. The concentrated extract was partitioned between EtOAc and H_2_O. The portion (20 mg) of the EtOAc extract (400 mg) was subjected to RP-HPLC (Cosmosil 5C_18_-AR-II, 10 mm i.d. × 250 mm, 40% acetonitrile with oven) to isolate theopederin K (**10**, 9.1 mg). The sponge was identified by one of us, YI.

All known compounds were identified by comparing its NMR and high-resolution mass spectroscopic (HRESIMS, Orbitrap) data with those published previously.

### Biological Tests

#### In Vitro Leishmanicidal Assay

The *leishmania* growth medium consisted of Medium-199 supplemented with 10% fetal calf serum, 100 IU/mL penicillin, and 100 µg/mL streptomycin. This medium was utilized for the cultivation of cutaneous-type promastigotes of *Leishmania major* (MHOM/SU/73/5ASKH). Promastigotes were cultured in a medium supplemented with heat-inactivated fetal bovine serum (10%) at 27 °C and 5% CO_2_ in an incubator.

The leishmanicidal effects of the extracts and isolated compounds were assessed using WST-8 method, as follows: cultured promastigotes were seeded at 5 × 10^4^/50 μL of medium per well in 96-well microplates. Then, 50 μL of varying concentrations of test compounds dissolved in a mixture of dimethyl sulfoxide (DMSO) and the medium, were added to each well. Each concentration was tested in triplicate. The microplate was then incubated at 27 °C in 5% CO_2_ for 48 h, following which 10 µL of the Cell Counting Kit-8 (Dojindo, Japan) was added to each well, and the plates were further incubated at 27 °C for 6 h. Optical density values were measured using an ARVO MX microplate reader (PerkinElmer Co., Ltd.), with a test wavelength of 450 nm and a reference wavelength of 595 nm.

The IC_50_ values were calculated by fitting the data to a non-linear regression using a dose–response inhibitory model in Microsoft Excel, with experiments conducted in triplicate (*n* = 3). Amphotericin B served as a positive control (IC_50_ < 0.1 µM).

### Cytotoxicity Assay

HepG2 and RAW264.7 cells were seeded in 96-well microplates at a density of 2.5 × 10^4^ cells/well. The following day, the cells were cultured with onnamide A for 2 d, and the cytotoxicity was determined using the WST-8 method.

## Results and Discussion

**Fig. 2:**
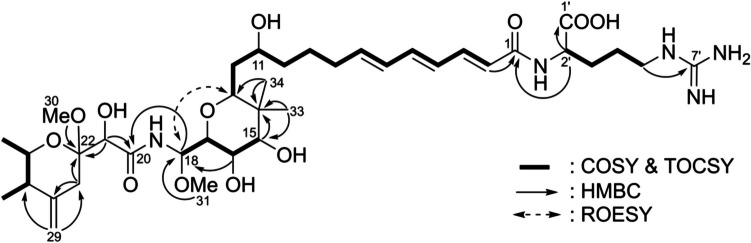
The key ^1^H–.^1^H COSY, TOCSY, HMBC, and ROESY correlations of onnamide G (**8**)

The molecular formula of onnamide G (**8**) was determined as C_38_H_63_N_5_O_12_ by HRESIMS (found for [M-H]^−^ *m/z* 780.439, calculated *m/z* 780.440, Δ −0.19 mmu), which is one carbon less than that of onnamide A (**1**). The ^1^H and ^13^C NMR spectra of **8** are almost superimposable on those of onnamide A (**1**), except for the signals corresponding to the positions around the two interconnected tetrahydropyran rings (Table 1) (Sakemi et al. 1988). The absence of the methoxy signal of the OCH_3_−32 bound to C-15 in **1**, and the higher-field chemical shift of C-15 (δ_C_ 77.0) in **8** than that in **1** (δ_C_ 80.6) suggests that the methoxy group is replaced by a hydroxy group at C-15 in **8**. Additionally, considering the absence of signals for the dioxymethylene at CH_2_−31 (δ_H_ 5.48/4.80) in **1** and the ten unsaturations in **8**, one degree less than that in **1**, onnamide G (**8**) likely possesses a bicyclic core structure with a hydroxyl group on CH-15, rather than the tricyclic core in **1**. This hypothesis was supported by the presence of an additional methoxy signal at CH_3_−31 (δ_H_ 3.37/δ_C_ 56.2) and the HMBC correlation from CH_3_−31 to the *N*-acyl hemiaminal methine at C-18(δ_C_ 84.7), as shown in Fig. 2. Following a detailed analysis of the NMR spectra, nine of the ten degrees of unsaturation, excluding the tetrahydropyran ring, were accounted for by the presence of an exomethylene tetrahydropyran ring (two degrees: exomethylene of C-24/29 with δ_C_ 147.9/109.9, a quaternary acetal carbon at C-22 with δ_C_ 100.8, and two secondary methyls at CH_3_−27/28), an amido group at C-20 (one degree: δ_C_ 173.5), an arginyl group (two degrees: carboxyl C-1′ with δ_C_ 179.8, guanidyl C-7′ with δ_C_ 159.0), and a conjugated carbonyl triene (four degrees: C-1–C-7). The conjugation of the carbonyl group with the triene is consistent with the strong UV absorption at 298 nm. The four partial structures from CH-2 to CH-13, CH-15 to CH-18, CH-25 to CH_3_−28, and from CH-2′ to CH_2_−5′of the arginyl group, were deduced by the key correlations of ^1^H–^1^H COSY and TOCSY, as shown in Fig. 2. Connectivities between each fragment were confirmed by the HMBC correlations from H-5′ to C-7′, H-2′ to C-1/1′, H-2 to C-1, H-33/34 to C-13/15, H-15 to C-14, H-18 to C-20, H-21 to C-20/22, H-23 to C-22/24, and H-29 to C-23/25. The ROESY signals between H-13 and H-18 confirmed the tetrahydropyran connection from C-13 to C-17, allowing us to elucidate the planar structure of onnamide G (**8**), as shown in Fig. 2.

**Table 1:** ^1^H and ^13^C NMR chemical shifts of onnamide G (**8**) and onnamide A (**1**) in methanol-*d*_4_

Position	^13^C		^1^H (*J* in Hz)	
	*	1	8	1^2^
1	168.4	168.3	-	-
2	123.9	124.4	6.08 (1H, d, 15.0)	6.07 (1H, d, 15.0)
3	141.8	141.9	7.15 (1H, dd, 15.0, 11.2)	7.13 (1H, dd, 15.0,11.2)
4	129.2	129.5	6.28 (1H, dd, 14.8, 11.2)	6.23 (1H, dd, 14.7, 11.3)
5	141.0	141.2	6.55 (1H, dd, 14.8, 10.8)	6.50 (1H, dd, 14.8, 10.7)
6	131.3	131.5	6.22 (1H, dd, 15.1, 10.8)	6.19 (1H, dd, 15.3, 10.7)
7	139.9	140.4	5.96 (1H, dt, 15.1, 7.3)	5.93 (1H, dt, 15.2, 6.9)
8	33.7	33.9	2.17–2.21 (2H, br m)	2.21 (1H, m)
				2.13 (1H, m)
9	25.6	26.1	1.50–1.55 (2H, br m)	1.59 (1H, m)
				1.40 (1H, m)
10	36.8	36.8	1.57–1.60 (2H, br m)	1.49 (1H, m)
				1.28 (1H, m)
11	70.9	71.0	3.78 (1H, m)	3.64 (1H, m)
12	37.3	37.3	1.55 (2H, m)	1.53 (2H, m)
13	78.7	78.7	3.81 (1H, dd, 8.5, 4.0)	3.47 (1H, dd, 8.1, 3.6)
14	40.5	42.2	-	-
15	77.0	80.6	3.68 (1H, d, 9.6)	3.62 (1H, d, 9.6)
16	70.7	75.5	3.85 (1H, dd, 9.6, 7.1)	4.16 (1H, dd, 9.7, 6.5)
17	76.4	70.8	3.98 (1H, dd, 7.1, 3.6)	3.98 (1H, dd, 9.3, 6.5)
18	84.7	74.9	5.46 (1H, d, 3.6)	5.79 (1H, d, 9.3)
20	173.5	174.3	-	-
21	73.7	74.0	4.24 (1H, s)	4.23 (1H, s)
22	100.8	101.3	-	-
23	34.3	34.8	2.40 (1H, brd, 14.3)	2.40 (1H, brd, 14.4)
			2.33 (1H, brd, 14.3)	2.32 (1H, brd, 14.4)
24	147.9	148.1	-	-
25	42.7	43.0	2.21 (1H, m)	2.18 (1H, m)
26	70.4	70.8	3.90 (1H, qd, 6.6, 2.5)	3.87 (1H, qd, 6.5, 2.4)
27	17.8	18.2	1.19 (3H, d, 6.6)	1.17 (3H, d, 6.5)
28	12.1	12.4	0.99 (3H, d, 6.8)	0.96 (3H, d, 6.9)
29	109.9	110.1	4.81 (1H, brs)	4.79 (1H, brs)
			4.66 (1H, brs)	4.63 (1H, brs)
30	48.5	48.8	3.26 (3H, s)	3.22 (3H, s)
31	56.2	87.6	3.37 (3H, s)	5.48 (1H, d, 6.9)
				4.80 (1H, d, 6.9)
32	-	61.9	-	3.55 (3H, s)
33	13.2	14.5	0.84 (3H, s)	0.85 (3H, s)
34	23.4	23.7	0.91 (3H, s)	1.00 (3H, s)
1'	179.8	179.0	-	-
2'	55.1	55.6	4.38 (1H, dd, 7.1, 5.4)	4.36 (1H, dd, 7.9, 5.3)
3'	31.1	31.2	1.89 (1H, m)	1.89 (1H, m)
			1.74 (1H, m)	1.75 (1H, m)
4'	25.8	26.3	1.62 (2H, m)	1.63 (2H, m)
5'	41.8	42.0	3.21 (2H, m)	3.19 (2H, m)
7'	159.0	158.7	-	-

* ^13^C chemical shifts of **8** were determined by HSQC and HMBC correlations

**Fig. 3:**
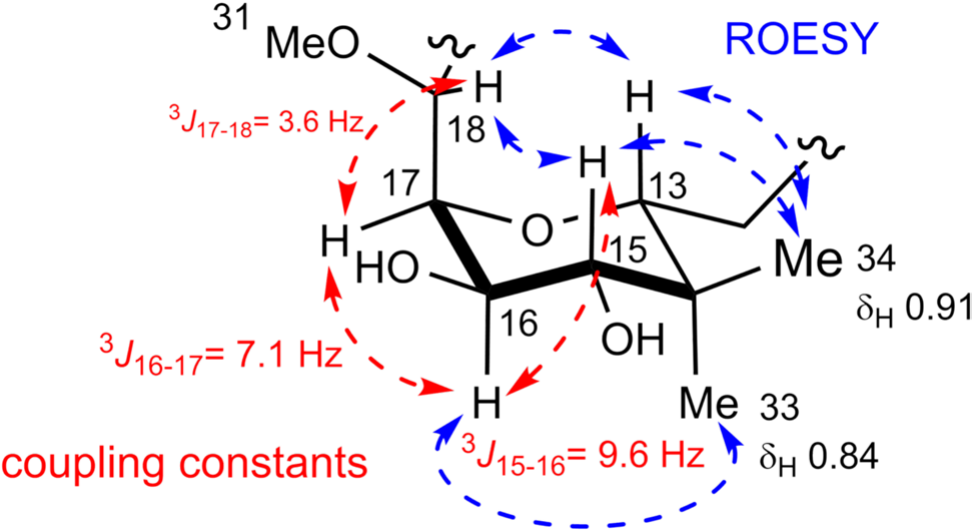
Key coupling constants and ROESY correlations on the tetrahydropyran moiety of onnamide G (**8**). The values of coupling constants and ROESY correlations are represented by red and blue dashed arrows, respectively

The geometry of the conjugated triene was assigned as all-*trans* based on its vicinal coupling constants (^3^*J*_H2-H3_ = 15.0 Hz, ^3^*J*_H4-H5_ = 14.9 Hz, ^3^*J*_H6-H7_ = 15.1 Hz). Because the ^1^H and ^13^C chemical shifts and coupling constants around C-22 to C-30 in **8** were almost identical to those of **1**, the stereochemistry of the tetrahydropyran ring around C-20 to C-30 was deduced to be the same as that of **1**. The relative stereochemistry of the tetrahydropyran ring from C-13 to C-18 was deduced from the coupling constants and ROESY correlations (Fig. 3). The large vicinal coupling constant between H-15 and H-16 (^3^* J* = 9.6 Hz) suggests that the CH-15 − CH-16 is in an antiperiplanar position, both being axial protons. This was supported by the ROESY correlations between CH_3_−34 and H-15/H-13, CH_3_−33 and H-16, H-18 and H-13/H-15, indicating that the tetrahydropyran ring has a chair conformation as shown in Fig. 3. The stereochemistry of C-21 and the Arg residue was presumed to be the same as that of **1**, based on the optical rotations (**1**: [α]_D_ + 99.1°, *c* 5.5, CH_3_OH, **8**: [α]_D_ + 35.8°, *c* 6.0, CH_3_OH), chemical shifts, and biosynthetic pathway (Matsunaga et al. 1992; Nakamura et al. 2023; Paul et al. 2002; Piel 2002; Sakemi et al. 1988; Shinde et al. 2007; Vuong et al. 2001).

The results of the leishmanicidal activity assay for compounds **1**–**10** are presented in Table 2. All compounds exhibited notable leishmanicidal activity, with 6,7-dihydro-onnamide A (**4**) showing the strongest activity (IC_50_ = 0.2 nM), followed by onnamide A (**1**; IC_50_ = 1.2 nM). These activities surpassed those of the existing drugs amphotericin B (IC_50_ 0.1 µM) and miltefosine (IC_50_ 8.3 µM).

**Table 2:** Leishmanicidal activity of the tested compounds

Compound	IC_50_ (µM) ± SD
Onnamide A (**1**)	0.0012 ± 0.00016
2*Z*-Onnamide A (**2**)	0.0038 ± 0.00030
6*Z*-Onnamide A (**3**)	0.0049 ± 0.00078
6,7-Dihydro-onnamide A (**4**)	0.0002 ± 0.00001
11-Oxo-Onnamide A (**5**)	0.0087 ± 0.0022
Onnamide B (**6**)	0.0065 ± 0.00016
Onnamide E (**7**)	0.14 ± 0.028
Onnamide G (**8**)	0.0063 ± 0.00033
Pseudoonnamide A (**9**)	0.022 ± 0.000060
Theopederin K (**10**)	0.030 ± 0.0038
Amphotericin B	0.063 ± 0.0043
Miltefosine	8.3 ± 0.12

Regarding the SAR, the structures of the most potent compounds, **1** and **4**, differ only in the reduction of the double bond in the side chain of the former. As hypothesized, the enhanced flexibility of the side chain increases its interaction with the target, resulting in stronger leishmanicidal activity of **4** than **1**.

2*Z*-Onnamide A (**2**), 6*Z*-onnamide A (**3**), onnamide B (**6**), and onnamide G (**8**) do not exhibit significant changes in activity regardless of the side-chain modifications. In contrast, pseudoonnamide A (**9**), in which the methoxy group of **1** is replaced with a hydroxyl group, exhibits a 20-fold decrease in activity. This suggests that the presence of an acetal group at C-22 of the pederic acid unit is essential for its activity. According to Tammam & El-Demerdash (Tammam and El-Demerdash 2023), in the context of anticancer activity, C-22 contributes more effectively when present as an acetal rather than as a hemiacetal group. Moreover, the marked decrease in the activity of onnamide E (**7**) indicates that the acetal structure at positions 18 and 16 plays a critical role in the anti-leishmania activity. However, as previously discussed, these groups do not contribute significantly to activity (Tammam and El-Demerdash 2023).

The anti-leishmania activity of theopederin K (**10**), a hydrolyzed onnamide analogue without the Arg residue, is reduced to 1/25 of that of **1**. Shinde et al*.* reported that the presence of an Arg group negatively affects the anticancer activity against a series of cancer cell lines (Shinde et al. 2007). However, the presence of the Arg residue had a positive impact on anti-leishmania activity*.*

According to a report by Hayashi et al. (Hayashi et al. 2024), the cytotoxicity (CC_50_) of onnamide A (**1**) and pseudoonnamide A (**9**) against Vero/TMPRSS2 cells is 7.34 µM and 4.00 µM, respectively. Additionally, in this study, the cytotoxicity of **1** against HepG2 cells (IC_50_ = 1.28 µM) and RAW264.7 cells (0.14 µM) resulted in a selectivity index (SI) of 1,067 and 117, respectively. This result meets the Walter Reed Army Institute for Research (WRAIR) and the Drugs for Neglected Diseases initiative (DNDi) criteria (Caridha et al. 2019) for as an SI > 5 folds.

Since the SAR for leishmania is not parallel to that for anticancer activity, the inhibitory mechanism against leishmania may differ from the anticancer mechanism, which has been attributed to protein synthesis inhibition (Lee et al. 2005). Based on these findings, we hypothesized that the anti-leishmania activity is associated with the disruption of *L. major* membrane.

**Fig. 4:**
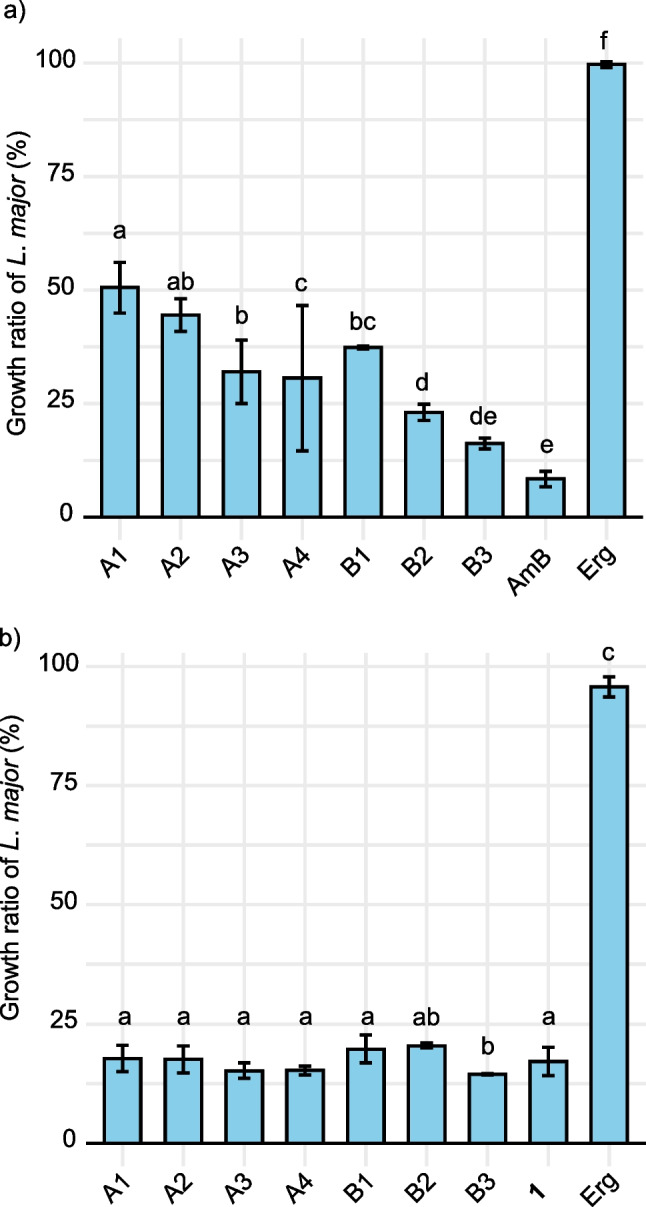
Growth ratio of *Leishmania major* (%) by amphotericin B (AmB) and onnamide A (1) in the presence or absence of ergosterol (Erg). **a**) A: AmB (100 nM) and Erg (A1 and A2: 200 nM, A3 and A4: 100 nM) were added simultaneously to the parasites. B: AmB (100 nM) and Erg (B1: 200 nM, B2: 100 nM, B3: 50 nM) were pre-mixed for 10 min before being added to the parasites. **b**) A: 1 (1 nM) and Erg (A1 and A2: 20 nM, A3 and A4: 10 nM) were added simultaneously to the parasites. B: 1 (1 nM) and Erg (B1: 20 nM, B2: 10 nM, B3: 5 nM) were pre-mixed for 10 min before being added to the parasites. Bars indicate mean ± SD, *n* = 3. Different letters indicate significant differences as determined by Tukey’s test (*p* < 0.05)

To gain insights into the inhibitory mechanism, onnamide A (**1**) was subjected to an affinity experiment with ergosterol (Erg), related to the mode of action of amphotericin B. Figure 4 illustrates the differential inhibition of *L*. *major* by amphotericin B (AmB) and onnamide A (**1**) in the presence of ergosterol. The high affinity of AmB for Erg is demonstrated by the reduced growth ratio when Erg is co-administered, as indicated by the decrease in the growth ratio shown in the rightmost bar of panel (a) (Fig. 4). This supports the conclusion that the mode of action of AmB involves interaction with Erg (Kumari et al. 2022). Notably, in the pre-mixed condition, the decrease in AmB activity is more stable and concentration-dependent. Erg alone does not exhibit any inhibitory effect, as indicated by the negligible growth ratio in the Erg-only bar.

In contrast, **1** retains its inhibitory effect even in the presence of Erg, as shown in panel (b). The results show that **1** activity remains stable under both simultaneous addition and pre-mixing conditions, with minimal concentration-dependent differences in growth ratios when mixed with Erg. This suggests that the mode of action of **1** is independent of interaction with Erg, distinguishing it from AmB. These findings highlight the mechanism of action for **1** that does not rely on its interaction with Erg, further differentiating **1** from the traditional leishmanicidal agent AmB.

Although the cell membrane of *L*. *major* contains episterol, a precursor of Erg, conducting experiments with episterol is challenging due to its high cost and limited availability. Therefore, Erg was used as a substitute in these experiments. The observed decrease in AmB activity under the pre-mixed conditions is consistent with its known interaction with membrane sterols and may reflect similar interactions with components of the *L*. *major* cell membrane.

In contrast, the activity of **1** appears to be largely unaffected by the presence of Erg under both simultaneous and pre-mixed conditions. These findings suggest that **1** may exert its leishmanicidal effect through a mechanism distinct from that of AmB, potentially independent of direct interaction with Erg or related sterols.

These findings highlight the potent leishmanicidal activity of onnamides identified in this study. Given their high selectivity indices, onnamides represent promising lead compounds for the development of new therapeutics against leishmaniasis. However, their complex molecular structures may pose challenges for chemical synthesis and large-scale production.

In 2014, the unculturable sponge symbiont *Candidatus Entotheonella* was identified as the bacterial producer of onnamide through a combination of single-cell analysis and metagenomic approaches (Piel 2002). As *Ca*. *Entotheonella* is known for its prolific biosynthesis of bioactive compounds, many researchers have attempted its cultivation, though without success to date. To address the issue of sustainable supply, ongoing efforts are focused on cultivating the source sponge as a viable alternative.

In 2017, labrenzin—an onnamide analogue lacking the *N*-acyl arginyl side chain—was isolated from the culturable bacterium *Labrenzia* sp. PHM005 (Schleissner et al. 2017). Interestingly, the genome of this bacterium encodes an onnamide-like biosynthetic gene cluster (Kačar et al. 2019), suggesting its potential utility in semi-synthetic production approaches. These microbial sources may offer practical platforms for future development and supply of onnamides.

## Conclusions

In conclusion, onnamides isolated from the marine sponge *Theonella* sp. exhibited potent in vitro leishmanicidal activity and high selectivity, indicating their strong potential as lead compounds for leishmanicidal drug development. Further investigations, including in vivo studies and mechanistic analyses, are essential to fully realize their therapeutic applicability.

## Supplementary Information

Below is the link to the electronic supplementary material.

Supplementary file1 (DOCX 374 KB)

## Data Availability

No datasets were generated or analysed during the current study.
