# Supplementary material for: Onnamides and a Novel Analogue, Onnamide G, as Potent Leishmanicidal Agents
Source: Mar Biotechnol (NY). 2025 Sep 5;27(5):132. doi: 10.1007/s10126-025-10494-1 (PMC12411591; doi:10.1007/s10126-025-10494-1)
Supplement: Supplementary file 1 — Supplementary file1 (DOCX 374 KB) [file 10126_2025_10494_MOESM1_ESM.docx]

**Onnamides and a novel analogue, onnamide G, as potent leishmanicidal agents**

Takahiro Jomori^1^*, Nanami Higa^1^, Trianda Ayuning Tyas^1^, Natsuki Matsuura^2^, Yudai Ueda^2^, Ayumi Suetake^2^, Shin Miyazaki^2^, Shuichi Watanabe^2^, Sei Arizono^3^, Yasuhiro Hayashi^3^, Ko Yasumoto^4^, Yuji Ise^5^, Toshiyuki Wakimoto^6^, Mina Yasumoto-Hirose^7^, Junichi Tanaka^1^, and Kanami Mori-Yasumoto^2^*

^1^Department of Chemistry, Biology and Marine Science, Faculty of Science, University of the Ryukyus, Nishihara, Okinawa 903-0213, Japan; tjomori7@cs.u-ryukyu.ac.jp

^2^Faculty of Pharmaceutical Sciences, Tokyo University of Science, Niijuku, Katsushika, Tokyo 125-8585, Japan; yasumoto@rs.tus.ac.jp

^3^Faculty of Agriculture, University of Miyazaki, 1-1 Gakuen-kibanadai-nishi, Miyazaki, Miyazaki 889-2192, Japan; hayashi_yasuhiro@cc.miyazaki-u.ac.jp

^4^Kitasato University School of Marine Biosciences, 1-15-1 Kitasato, Minami, Sagamihara, Kanagawa 252-0373, Japan, yasumoto@kitasato-u.ac.jp

^5^ Faculty of Human Environmental Studies, Hiroshima Shudo University, Ozuka, Asaminami-ku, Hiroshima 731-3195, Japan; libertador0429@gmail.com

^6^Faculty of Pharmaceutical Sciences, Hokkaido University, Sapporo, Hokkaido 060-0812, Japan; wakimoto@pharm.hokudai.ac.jp

^7^Tropical Technology Plus, Uruma, Okinawa 904-2234, Japan; myhiro@ttc.co.jp

* Corresponding authors.

E-mail address: tjomori7@cs.u-ryukyu.ac.jp (T. Jomori), yasumoto@rs.tus.ac.jp (K. Mori-Yasumoto)

**Supporting information**

Table of Contents page

Figure S1. ^1^H NMR spectrum of onnamide G (**8**) in MeOD (500 MHz) 3

Figure S2. HSQC spectrum of onnamide G (**8**) in MeOD (500 MHz) 4

Figure S3. HMBC spectrum of onnamide G (**8**) in MeOD (500 MHz) 5

Figure S4. ^1^H–^1^H COSY spectrum of onnamide G (**8**) in MeOD (500 MHz) 6

Figure S5. TOCSY spectrum of onnamide G (**8**) in MeOD (500 MHz) 7

Figure S6. ROESY spectrum of onnamide G (**8**) in MeOD (500 MHz) 8

Figure S7. Negative HRESIMS data of onnamide G (**8**) in MeOD (500 MHz) 9

Figure S8. FTIR spectrum of onnamide G (**8**) in MeOD (500 MHz) 9

Figure S9. UV spectrum of onnamide G (**8**) in MeOD (500 MHz) 9


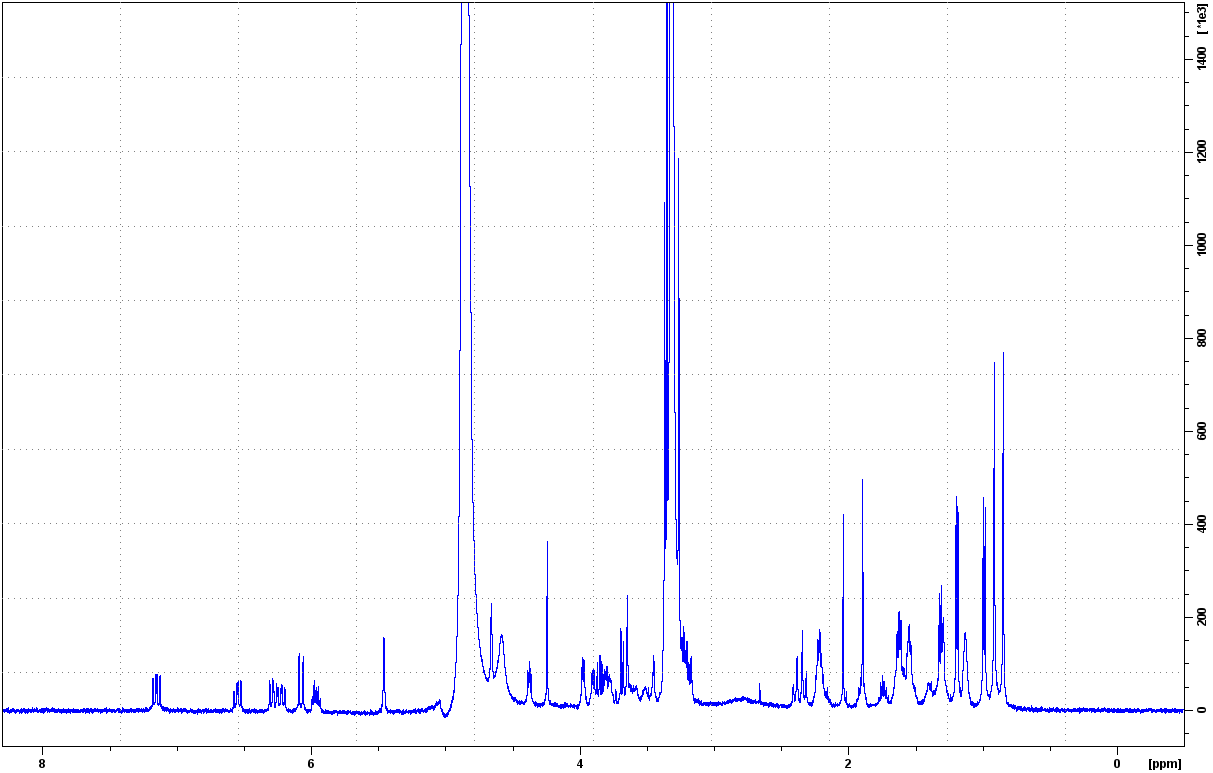


**Figure S1**. ^1^H NMR spectrum of onnamide G (**8**) in methanol-*d*_4_ (500 MHz).

**a)**


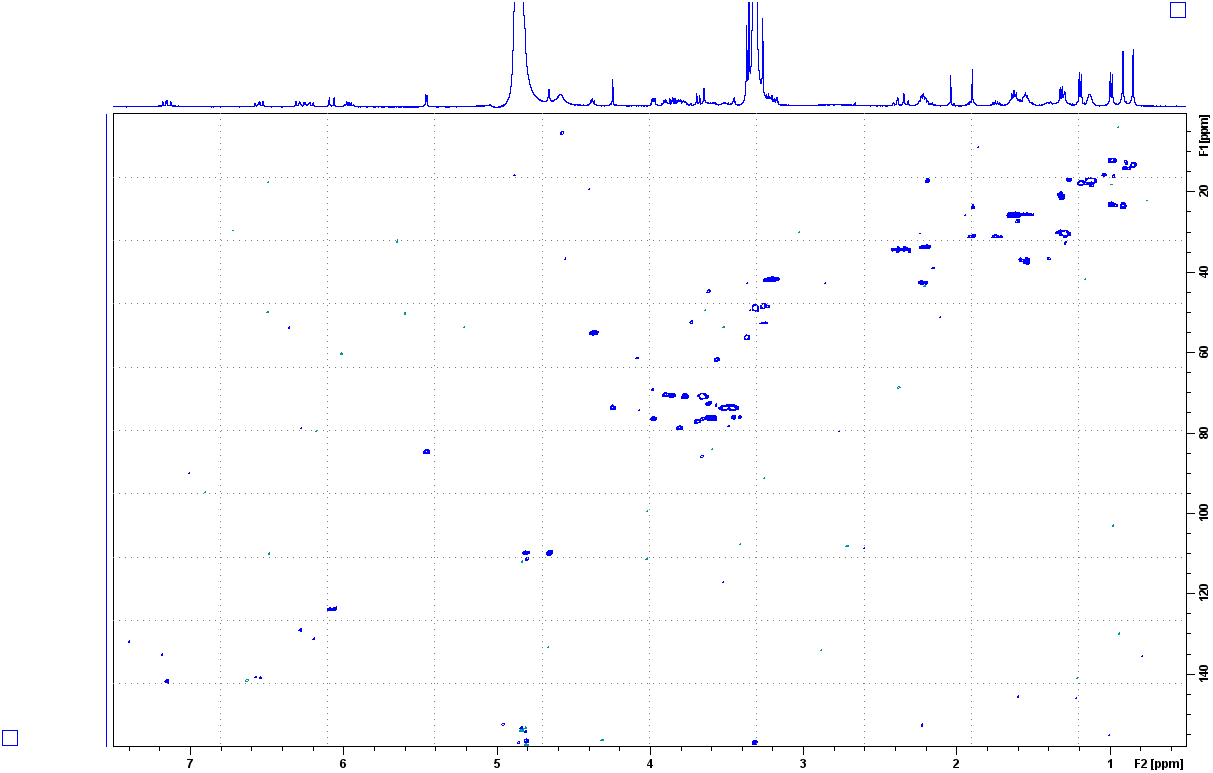


**b)**


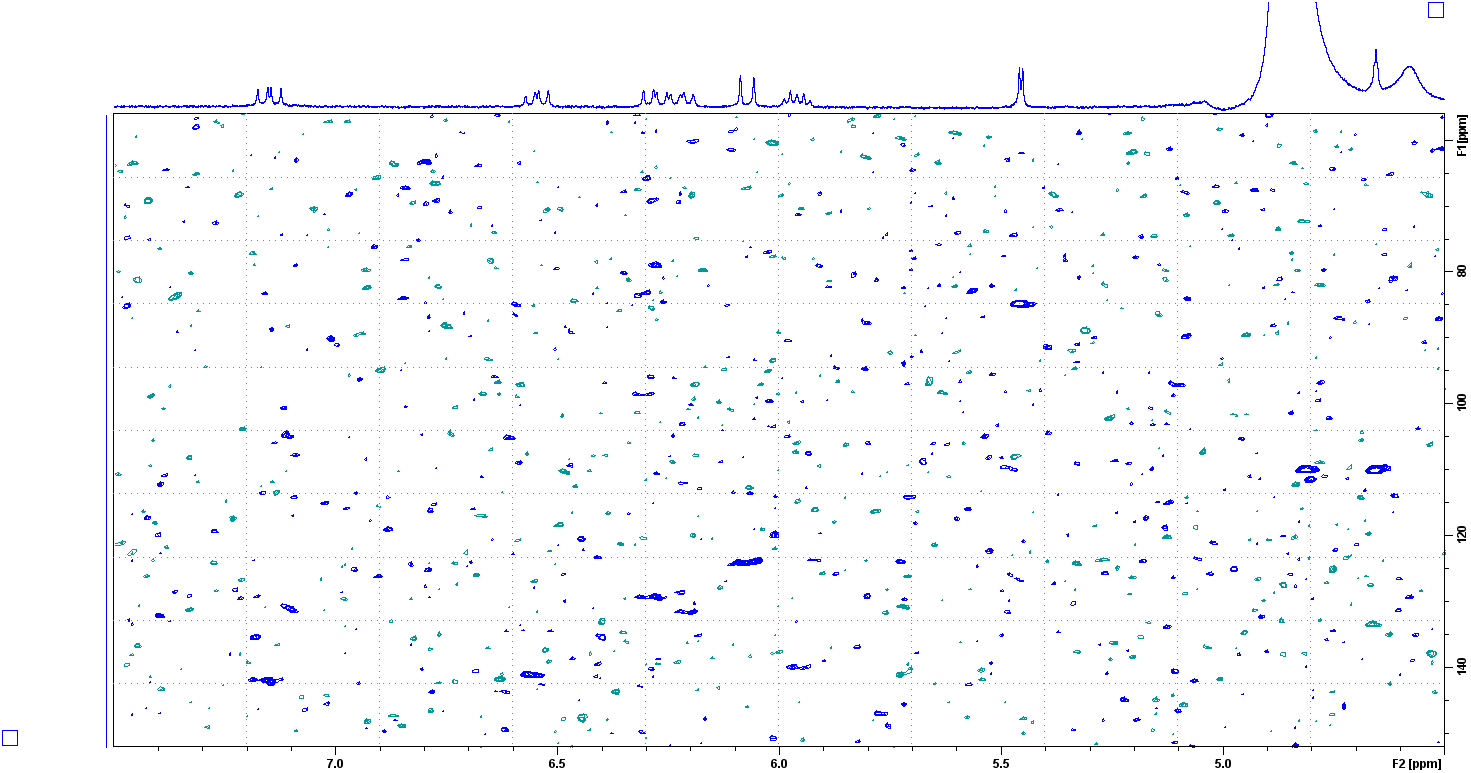


**Figure S2**. HSQC spectrum of onnamide G (**8**) in methanol-*d*_4_ (500 MHz).

a) Whole spectrum. b) Enlarged spectrum from 4.5 to 7.5 ppm with higher intensity.


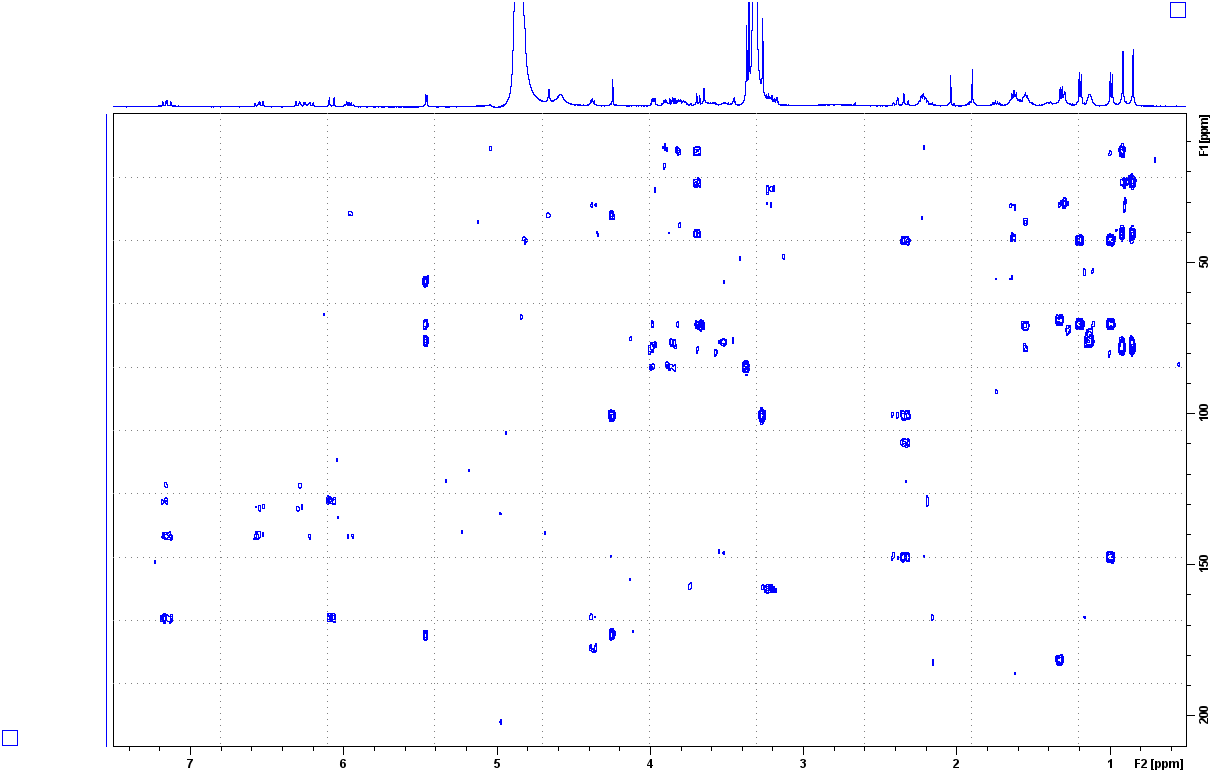


**a)**

**b)**

H-3'


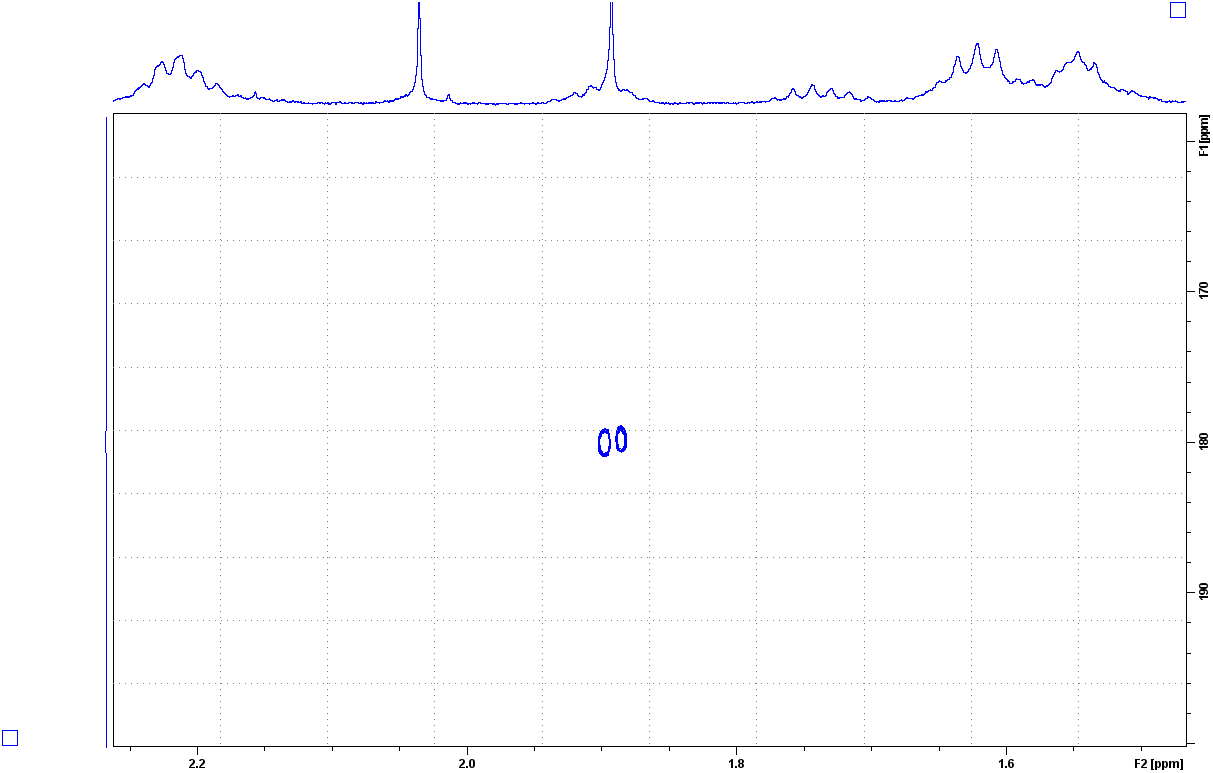


C-1'

179.8 ppm

**Figure S3**. HMBC spectrum of onnamide G (**8**) in methanol-*d*_4_ (500 MHz).

HMBC spectra were measured with following condition to observed cross peaks.

a) CNST [13] parameter were set to “7” and b) “10” which is default value.


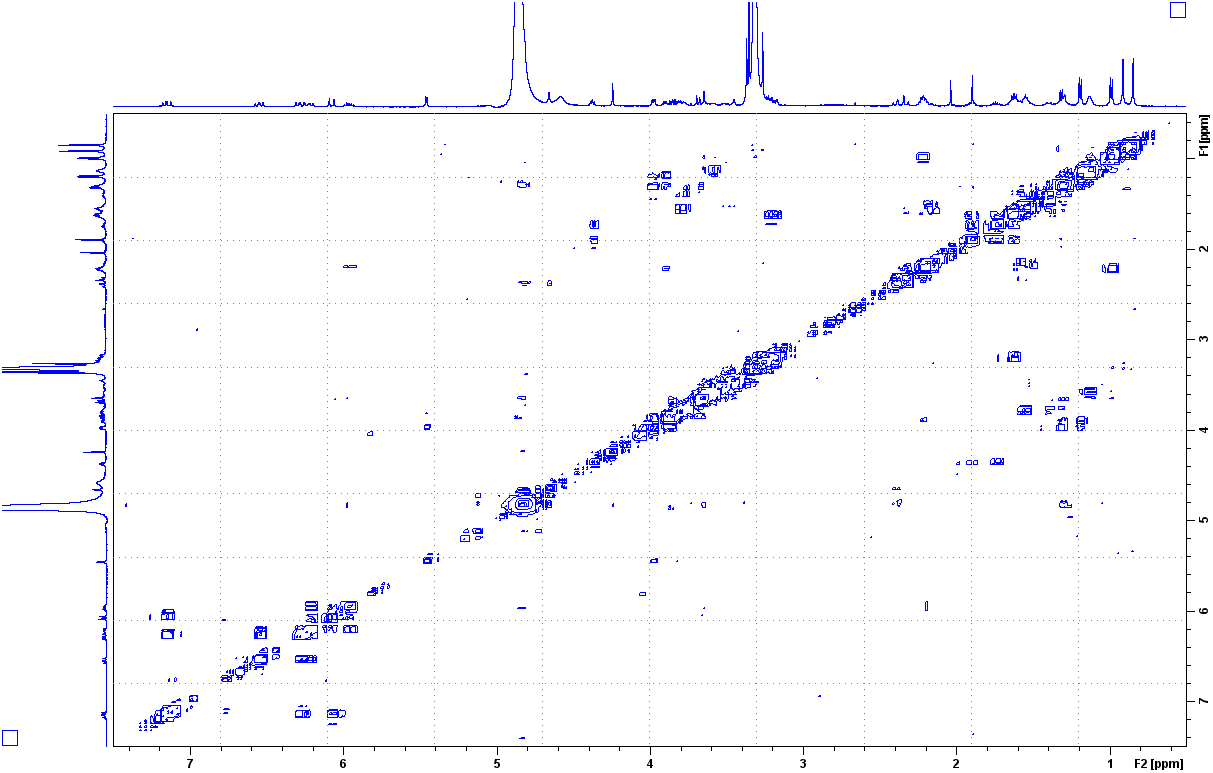


**Figure S4**. ^1^H–^1^H COSY spectrum of onnamide G (**8**) in methanol-*d*_4_ (500 MHz).


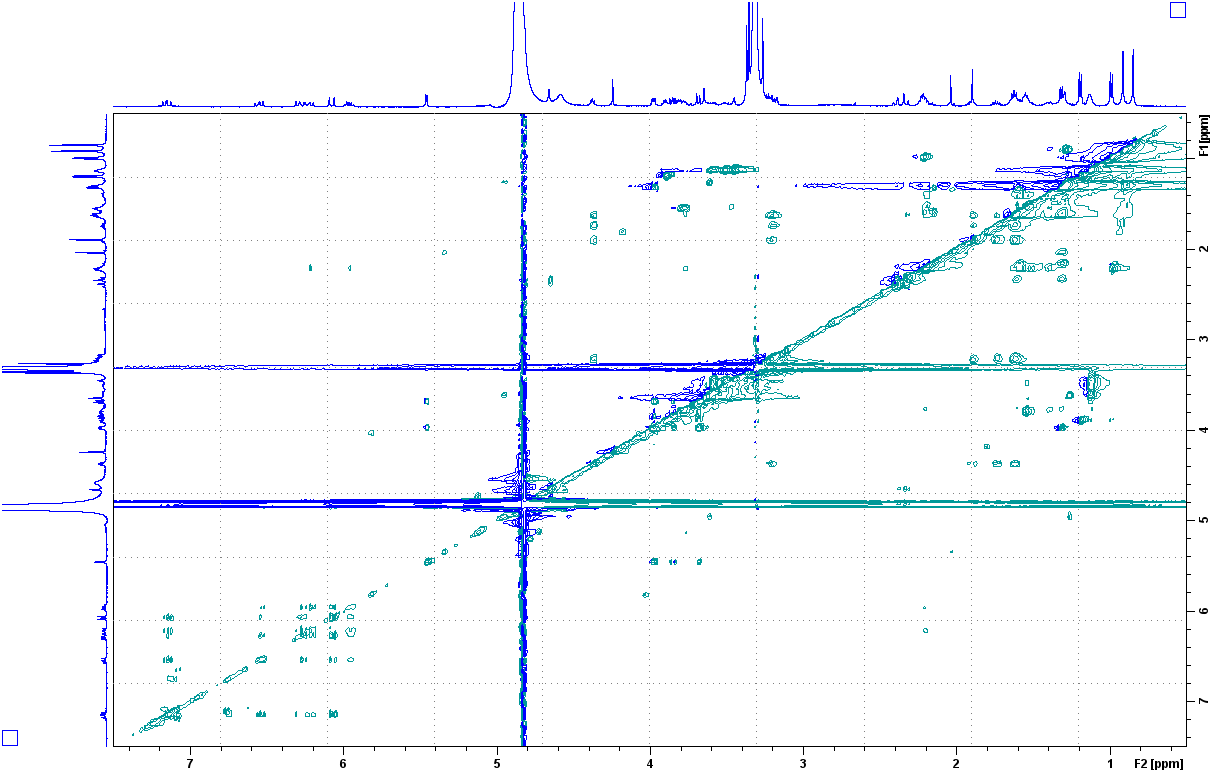


**Figure S5**. TOCSY spectrum of onnamide G (**8**) in methanol-*d*_4_ (500 MHz).


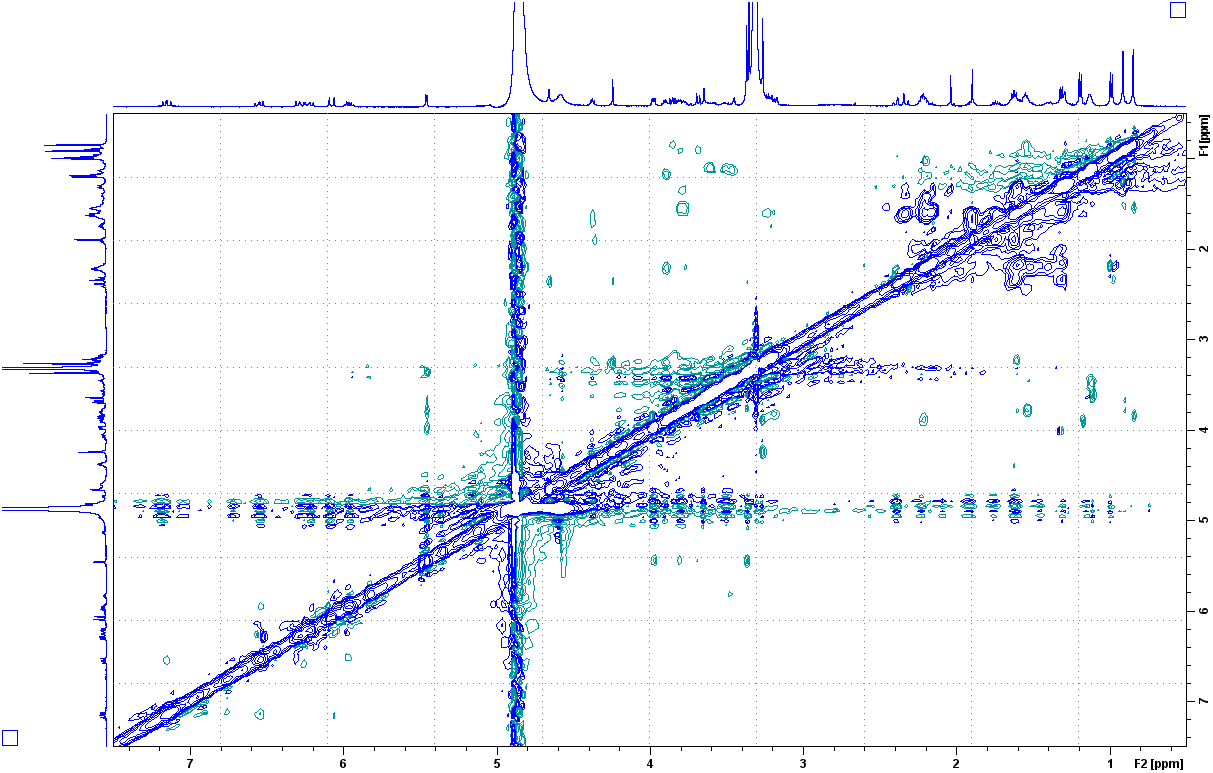


**Figure S6**. ROESY spectrum of onnamide G (**8**) in methanol-*d*_4_ (500 MHz).


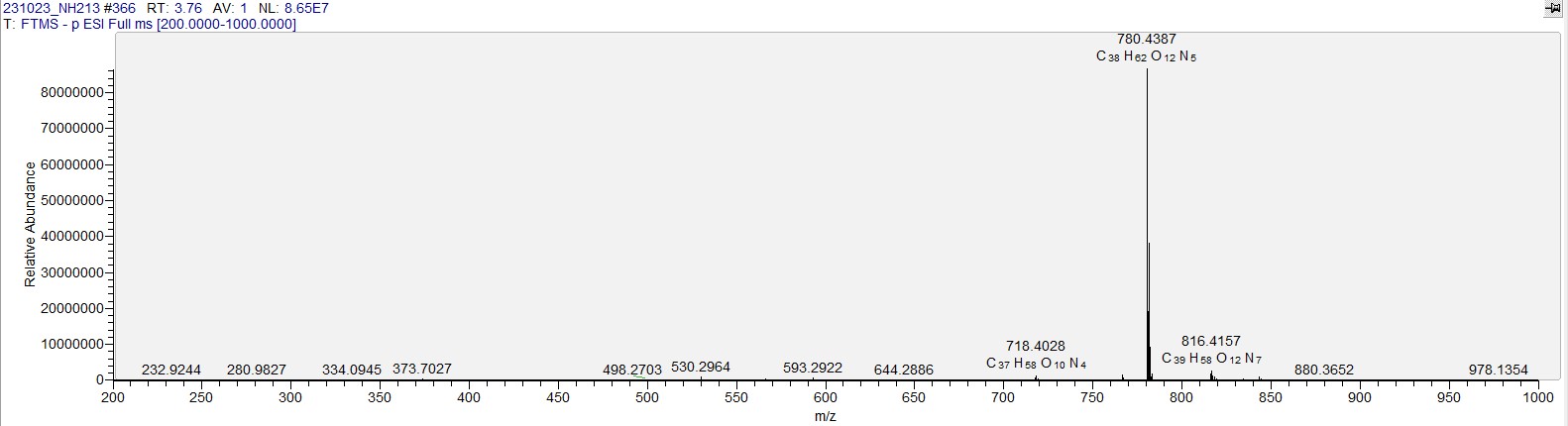


**Figure S7**. Negative HRESIMS data of onnamide G (**8**).


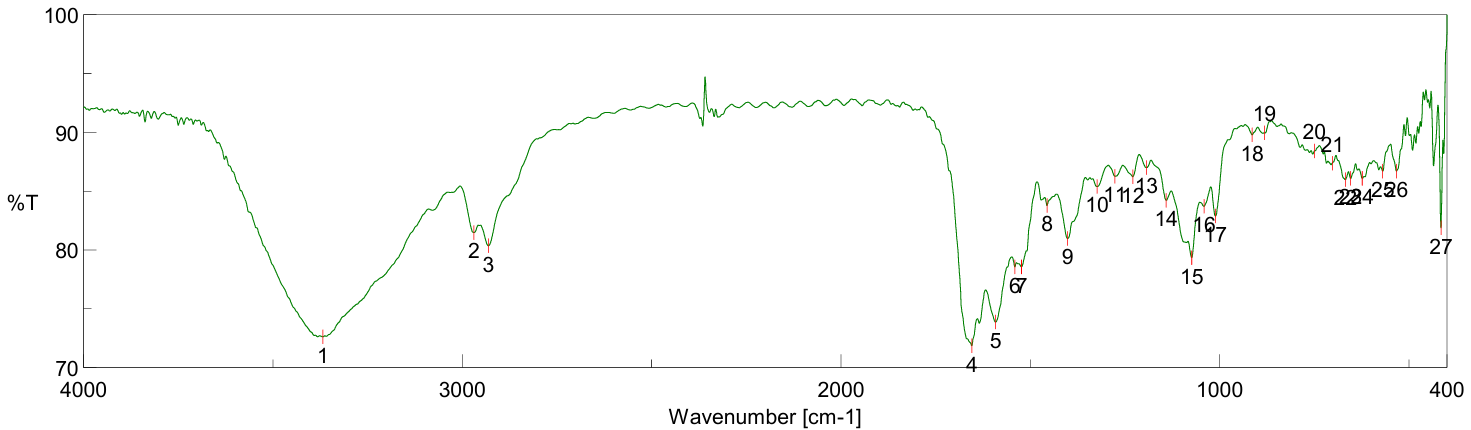


**Figure S8**. FTIR spectrum of onnamide G (**8**).
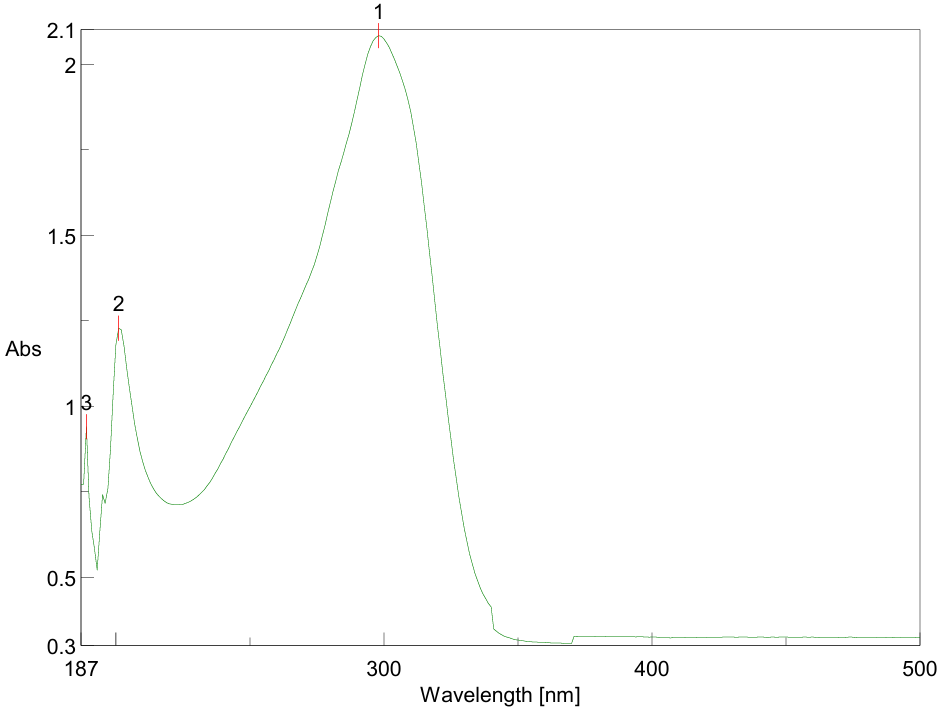


**Figure S9**. UV spectrum of onnamide G (**8**).
